# Systemic Sclerosis-Specific Antibodies: Novel and Classical Biomarkers

**DOI:** 10.1007/s12016-022-08946-w

**Published:** 2022-06-18

**Authors:** Ilaria Cavazzana, Tamara Vojinovic, Paolo Airo’, Micaela Fredi, Angela Ceribelli, Eleonora Pedretti, Maria Grazia Lazzaroni, Emirena Garrafa, Franco Franceschini

**Affiliations:** 1 Rheumatology and Clinical Immunology Unit, ASST Spedali Civili, piazzale Spedali Civili 1, Brescia, 25123 Italy; 2 Department of Clinical and Experimental Sciences, University of Brescia, Brescia, Italy; 3 Division of Rheumatology and Clinical Immunology, Humanitas Research Hospital IRCCS, Rozzano, Milan Italy; 4 Department of Biomedical Sciences, Humanitas University, Pieve Emanuele, Milan Italy; 5 Department of Laboratory Diagnostics, ASST Spedali Civili, Brescia, Italy

**Keywords:** Systemic sclerosis, Disease-specific autoantibodies, Anti-nuclear antibodies

## Abstract

Disease-specific autoantibodies are considered the most important biomarkers for systemic sclerosis (SSc), due to their ability to stratify patients with different severity and prognosis. Anti-nuclear antibodies (ANA), occurring in subjects with isolated Raynuad’s phenomenon, are considered the strongest independent predictors of definite SSc and digital microvascular damage, as observed by nailfold videocapillaroscopy. ANA are present in more than 90% of SSc, but ANA negativity does not exclude SSc diagnosis: a little rate of SSc ANA negative exists and shows a distinct subtype of disease, with less vasculopathy, but more frequent lower gastrointestinal involvement and severe disease course. Anti-centromere, anti-Th/To, and anti-Topoisomerase I antibodies could be considered as classical biomarkers, covering about 60% of SSc and defining patients with well-described cardio-pulmonary complications. In particular, anti-Topoisomerase I represent a risk factor for development of diffuse cutaneous involvement and digital ulcers in the first 3 years of disease, as well as severe interstitial lung disease (ILD). Anti-RNA polymerase III is a biomarker with new clinical implications: very rapid skin thickness progression, gastric antral vascular ectasia, the occurrence of synchronous cancers, and possible association with silicone breast implants rupture. Moreover, novel SSc specific autoantibodies have been globally described in about 10% of “seronegative” SSc patients: anti-elF2B, anti-RuvBL1/2 complex, anti-U11/U12 RNP, and anti-BICD2 depict specific SSc subtypes with severe organ complications. Many autoantibodies could be considered markers of overlap syndromes, including SSc. Anti-Ku are found in 2–7% of SSc, strictly defining the PM/SSc overlap. They are associated with synovitis, joint contractures, myositis, and negatively associated with vascular manifestation of disease. Anti-U3RNP are associated with a well-defined clinical phenotype: Afro-Caribbean male patients, younger at diagnosis, and higher risk of pulmonary hypertension and gastrointestinal involvement. Anti-PM/Scl define SSc patients with high frequency of ILD, calcinosis, dermatomyositis skin changes, and severe myositis. The accurate detection of autoantibodies SSc specific and associated with overlap syndromes is crucial for patients’ stratification. ANA should be correctly identified using indirect immunofluorescent assay and a standardized way of patterns’ interpretation. The gold-standard technique for autoantibodies’ identification in SSc is still considered immunoprecipitation, for its high sensitivity and specificity, but other assays have been widely used in routine practice. The identification of SSc autoantibodies with high diagnostic specificity and high predictive value is mandatory for early diagnosis, a specific follow-up and the possible definition of the best therapy for every SSc subsets. In addition, the validation of novel autoantibodies is mandatory in wider cohorts in order to restrict the gap of so-called seronegative SSc patients.

## Introduction

Systemic sclerosis (SSc), also known as scleroderma, is an immune-mediated rheumatic disease with significant clinical heterogeneity associated with high morbidity and mortality [1]. Because of SSc extremely changing course with regard to the clinical characteristics and evolution, establishing the best management and the most effective treatment is very challenging. Consequently, the recognition of biomarkers and the search for new disease biomarkers is extremely useful.

Biomarker definition was established by a joint task of U.S. Food and Drug Administration and the National Institutes of Health as “a defined characteristic that is measured as an indicator of normal biological processes, pathogenic processes, or responses to an exposure or intervention, including therapeutic interventions” [2]. They are by definition objective, quantifiable characteristics of biological processes and can derive from molecular, histological, radiographic, or physiological characteristics [3]. Biomarkers are critical to the rational development of drugs and medical devices [4]. Based on their applications and utility as surrogate endpoint, there are different subtypes of biomarkers. Furthermore, a single biomarker can meet multiple criteria for different uses [3]. A perfect example of such biomarker are autoantibodies.

Disease-specific autoantibodies are important for determination of different clinical groups of SSc by stratifying patients in more homogeneous subsets. There is a pressing need for better diagnostic distinction and evaluation of SSc patients and thus for further studies of potential biomarkers as diagnostic, disease severity, and prognostic tool.

Serum autoantibodies directed against multiple intracellular antigens are the serological hallmark of SSc: they are detectable in more than 95% of patients and characterized by almost nine SSc-specific autoantibodies directed against nuclear or nucleolar autoantigens. The most frequent autoantibodies are anti-topoisomerase I (anti-TopoI), anti-centromere (ACA), and anti-RNA polymerase III (antiRNAP3), while anti-Th/To, anti-fibrillarin, and anti-NOR90 are more rarely found [1]. These antibodies are relatively specific for SSc, but individually, they are only moderately to weakly sensitive. Other autoantibodies targeting PM/Scl proteins (PM/Scl-100 and PM-Scl-75), Ro52 (also called TRIM21), or Ku are not specific to SSc and are also found in other systemic autoimmune diseases [5].

The co-existence of two different SSc-specific autoantibodies is a very rare event, so they are considered mutually exclusive [6], identifying some clinical clusters [7] with different prognosis [8]. Furthermore, they are present at disease onset, their titer is quite stable during the SSc course [9], so they represent a true diagnostic tool in clinical practice.

Few data are available regarding their predictive value: preliminary data suggest the occurrence of SSc-specific antibodies in about 50% of patients, years before clinical SSc diagnosis. In particular, they are detectable in 75% of SSc renal crisis (SRC) and 40% of SSc without renal complication [10].

Although their pathogenetic role has not been definitely elucidated, recently, other authors have shown that immunocomplexes containing SSc-specific antibodies are able to activate in vitro endothelial cells and fibroblasts, suggesting a possible active role in inducing a pro-inflammatory and profibrotic effect [11, 12].

## Methodological Aspects Related to the Identification of SSc Autoantibodies

Among SSc-specific antibodies, only three of them are currently identified by routine methods and are included in the SSc classification criteria as described [13]. In particular, anti-TopoI and ACA have been used for about 30 years for SSc diagnosis, while antiRNAP3 antibodies were added to routine screening only recently [14–16]. Several additional autoantibodies, namely anti-Th/To and anti-U3RNP, fairly specific for SSc, are associated with unique clinical features (limited or diffuse cutaneous involvement) and are useful in predicting clinical manifestations of SSc [17, 18]. Nevertheless, their detection is restricted to some specialty laboratories, due to the limited availability of commercial tests [14, 19] and some technical issues, represented by a limited sensitivity of these immunoassays [1, 20].

The technical approach for the identification of SSc autoantibodies is crucial as it is linked to the correct identification of specificities: the gold-standard technique for autoantibodies’ identification in SSc patients is still considered the immunoprecipitation (IP) technique, for its high sensitivity and specificity, but it is a time- and labor-consuming method. Thus, additional routine techniques have recently been developed to allow an easier and reliable identification of autoantibodies in most laboratories worldwide.

### IP

Immunoprecipitation (IP), using urea-polyacrylamide gel electrophoresis (PAGE) analysis of the RNA components (RNA-IP) by silver staining or by using ^32^P-labeling of cells (protein-IP), is the standard method for identifying antibodies directed towards ribonucleoprotein or protein antigens, respectively. It is performed only in a small number of research laboratories and for several autoantibodies for which no widely available validated immunoassay kit has yet been produced [21].

### IIF

Despite the well-known limitations of indirect immunofluorescence (IIF), such as to be time-consuming method with the need of expertise for result interpretation, it is currently recommended as the gold-standard method for ANA screening and is still the preferred screening immunoassay for detection of the majority of clinically relevant SSc autoantibodies [22–24]. The International Consensus on ANA pattern (ICAP) has established the use of human epithelial type 2 (HEp-2) cells, originating from human laryngeal cancer, as substrate for IIF, in order to obtain information on the staining pattern and the antibody titer, with the advantage of high sensitivity. To overcome labor-intensive and time-consuming clinical work-up procedures and to minimize the variation of pattern and titer estimates, automated reading systems have been proposed. Furthermore, recent studies have demonstrated agreement with conventional IIF interpretation in some cases with a hit rate close to 100% in samples with a clearly negative or positive ANA tests result [22, 24, 25]. However, automated pattern recognition is limited to a few main IIF patterns, to date, and not used in routine practice.

The most typical SSc pattern recognized by IIF is ACA, very specific for SSc, as well as two other patterns that may be suggestive for SSc: the nucleolar pattern, usually indicative of anti-U3 RNP, anti-Th/To, anti-NOR 90, and anti-PM/Scl antibodies, and the speckled pattern that may be related to the presence of anti-TopoI, anti-RNAP3, or other less-frequently reported antibodies such as anti-U11/U12 RNP, anti-Ku, anti-U1 RNP, and anti-RuvBL1/2, characterized by peculiar nucleolar, nuclear, and metaphase staining https://www.anapatterns.org). Recently, an observational retrospective study including 608 patients with a confirmed rare ANA pattern detected by IIF on HEp-2 cells showed that rare cytoplasmic patterns were frequently associated to SSc as a unique finding with no SSc-specific antibodies [26], confirming that the IIF technique remains a valid tool to identify SSc cases.

When ANA patterns are identified in SSc patients, they need to be further characterized by using different techniques, mostly represented by ELISA and IB in most laboratories worldwide, together with chemiluminescence immunoassay (CIA), fluorescence enzyme immunoassay (FEIA), and Multiplex immunoassay, since IP is available only in a few centers worldwide.

### ELISA

Enzyme-linked immunosorbent assay (ELISA) could be used as an alternative to IIF for ANA screening, particularly in laboratories which conduct a large number of tests with full automation, for several simultaneous analyses. However, the comparison of sensitivity and specificity for ELISA and IIF is still being discussed, since a negative IIF result does not exclude the presence of antibodies, thus it is reasonable to use ELISA to confirm IIF ANA result.

ELISA has replaced the obsolete old techniques (such as immunodiffusion or counterimmunoelectrophoresis), since they were technically difficult assays especially when several samples needed to be tested simultaneously. Together with the classical commercialized ELISA for SSc (like the one used to test anti-RNAP3, anti-TopoI, ACA, anti-Ro52) several other ELISA are under evaluation, such as the ELISA for differentiating two isotypes of anti-PM/Scl antibodies, that could be related to different SSc clinical subset [27].

### IB

Immunoblotting (IB) is a powerful technique that simultaneously can assess different autoantibodies in SSc patients with the advantage of cost-effectiveness, compared to ELISA used for detection of a single autoantibody. However, some limitations have to be considered: protein degradation, during the antigen source preparation, could elicit in low sensitivity in some antibodies’ detection (i.e., topoisomerase I and centromere), difficulty of validation process for rare specificities and the lack of quality management programs for rare SSc-specific autoantibodies. IB has been proposed for stratification of SSc based on the use of specific and associated autoantibodies [28], identifying five major autoantibodies’ clusters with specific clinical and serological associations in a cohort of 505 Australian SSc patients [7]. They suggest that using autoantibodies profile for sub classification and stratification will improve prognosis, disease management and help to identify SSc patients for a personalized therapeutic approach.

## ANA in SSc

Although the current SSc criteria [13] do not include the presence of ANA, the detection ANA and SSc-associated antibodies may be a valuable tool in the diagnosis of patients with very early SSc [29] or only carrying Raynaud’s phenomenon [30]. A large prospective, single-center study of patients with rigorously defined isolated Raynaud’s phenomenon demonstrated that the presence of ANA and SSc-associated antibodies were the strongest independent predictor of definite SSc [30]. Moreover, the occurrence of ANA, as well as SSc-specific autoantibodies, predicted the evolution of nailfold videocapillaroscopy findings during time. So, they could be considered the biomarkers of development of severe microvascular damage in patients carrying isolated Raynaud’s phenomenon [30]. ANA negativity does not exclude SSc diagnosis: a little rate of SSc ANA negative exists and shows a distinct subtype of disease, with less vasculopathy, a greater proportion of men, more frequent lower gastrointestinal involvement, and a more severe disease course [31].

### Prognostic Value SSc-Specific Antibodies

As previously mentioned, few data are available regarding the occurrence of SSc-specific antibodies before the clinical diagnosis of SSc. Different authors described the high predictive value of ACA, anti-TopoI, or anti-Th/To antibodies in patients with isolated Raynaud’s phenomenon [28, 30, 32]. Anyway, the significance of SSc-specific autoantibodies in absence of clinical features of the disease is still underaddressed. Some authors reported the occurrence of circulating anti-Ro52, anti-Ro60, and ACA decades before disease manifestations, until the formal SSc diagnosis. By contrast, anti-RNAP3 and anti-TopoI autoantibodies rise over time and become elevated only few years prior to clinical disease [10].

## Classical SSc-Specific Markers

### ACA

Anti-centromere antibodies (ACA) are found in about 30% of all SSc patients with higher frequency in Caucasian than African American or Asian cohorts [33]. ACA are mainly directed towards three centromere proteins, namely CENP-A, B, and C: CENP-B, considered the major epitope, is 80-kDa deoxyribonucleic acid (DNA)-binding protein via its N-terminal region. CENP-B is located in the central part of the kinetochore of the centromere and consists of several major and minor epitopes, corresponding to different biologically functional regions [34]. Other centromere proteins have been identified, such as CENP-D, E, F, H, and O [35], but these reactivities are not specific for SSc. More recently protein microarray assays identified other CENP-family autoantigens that could be relevant in some subtypes of SSc: in fact, antibodies to CENP-P and Q could identify some SSc with interstitial lung disease (ILD) or renal disease [36].

ACA are considered one of the serological markers of limited cutaneous (lcSSc), characterized by long-lasting Raynaud’s phenomenon, followed by years of progressive skin thickening. The cutaneous involvement of ACA+ SSc is usually characterized by dermal thickness of hands and/or feet distally from elbow and knee, respectively [37]. ACA is not only considered a diagnostic marker of SSc but also a predictive factor of future development of the disease if found in patients with Raynaud’s phenomenon and/or nailfold capillary abnormalities [28].

ACA+ SSc patients rarely show diffuse cutaneous involvement [38], finger ulcers, digital tuft resorption, or finger contractures [39]. The diffuse cutaneous SSc (dcSSc) is estimated in 2–7% of ACA+ patients and the peak extension of dermal induration (measured by Rodnan skin score) occurs later than in other diffuse SSc groups; nevertheless, ACA+ dcSSc shows the same clinical picture as other dcSSc [38, 40].

ACA are frequently associated with cutaneous calcinosis, due to deposition of insoluble calcium salts in the skin and subcutaneous tissue, usually occurring over the pressure points. Cutaneous calcinosis often develops in a subgroup of patients with lcSSc and Raynaud’s phenomenon, lower sphincter esophageal dysfunction, sclerodactyly, and telangiectasia. These cases were previously defined by an acronym as CREST-syndrome, now subsumed under lcSSc [41]. ACA are strictly associated with pulmonary hypertension (PAH), not related to ILD [40], although no association between survival and autoantibodies’ status was demonstrated [42]. PAH is estimated to occur in about 10–20% of ACA+ SSc patients [43], but ACA+ patients show reduced DLCO values even without a formal diagnosis of PAH, consistent with indolent microvascular damage and increased vascular pulmonary resistance [44]. Analyzing the risk factors contributing to the progression of PAH in SSc, the occurrence of ACA is significantly associated with a rise of pulmonary arterial systolic pressure more than 2.5 mmHg per year (odds ratio (OR) of 8.7). So, if overall survival in SSc patients with ACA is better than in those without, PAH is the major cause of death in this subset [43].

### Anti-Topo I Antibodies

Known since 1979 as anti-Scl70 [45], anti-topoisomerase I (anti-TopoI) antibodies recognize a nuclear protein of 70–100 kD, clustered with DNA molecule and implicated in DNA chain conformation changing during cellular replication [46]. In particular, topoisomerase enzymes change the tertiary structure of the DNA molecule, relaxing supercoiled DNA through breaking and rejoining one strand at a time (type I enzymes) or by catalyzing catenation/decatenation of DNA rings (type II enzymes) [47, 48]. Anti-TopoI antibodies occur almost exclusively in SSc, while anti-TopoIIα antibodies have been reported in different autoimmune disorders [30]. Different epitopes are recognized by anti-TopoI antibodies, but the immunodominant site is located in the 489–573 amino acid sequence [49].

The TopoI antigen is selectively expressed in cells during S, G2 phase and metaphase of eucaryotic cell cycle [50]. Anti-TopoI antibodies show a typical fluorescent speckled pattern of nuclei with nucleolar and mitosis staining, coded as AC29, according to International Consensus ANA pattern (ICAP) nomenclature [51].

Anti-TopoI antibodies are highly specific for SSc and represent a predictive factor for disease development when found in patients with isolated Raynaud’s phenomenon [30].

Different studies reported a prevalence of 30–70% in diffuse cutaneous type of SSc [13] with a comparable rate between Caucasian [52], African American [53, 54], and Japanese cohorts [55, 56].

Anti-TopoI are associated with diffuse cutaneous involvement with ischemic digital ulcers [45], flexion contractures in metacarpophalangeal and proximal interphalangeal joints [57], and hand disability [58]. Anti-TopoI represent a risk factor for development of diffuse cutaneous involvement and digital ulcers in the first 3 years of disease, according to a prospective longitudinal European database [59].

When compared with ACA+ SSc, patients with anti-TopoI+ showed a lower interval between Raynaud’s onset and the development of a new non-Raynaud symptom, higher rate of synovitis, muscle involvement (namely, weakness, atrophy, or overt myositis), heart conduction blocks, ILD, restrictive lung defect, and dyspnea [40]. PAH is frequently found in anti-TopoI+ SSc but secondary to pulmonary fibrosis [40].

ILD is the most severe organ manifestation in SSc, and it is a leading cause of mortality [60], strictly associated with anti-TopoI occurrence, independently from the extension of cutaneous involvement [61]. Different cohorts, analyzing the prevalence and severity of ILD in SSc, recognize anti-TopoI positivity as one of the risk factors for the development of SSc-ILD [62, 63], as well as their presence being an indicator of unfavorable prognosis [40]. Anti-TopoI are globally correlated with fibrosis in SSc: some authors report that anti-TopoI positive SSc patients show lower FVC and DLCO values, as well as higher Rodnan Skin Score (RSS) values, compared with negative subjects [64]. The titer of anti-Topo I autoantibody correlates with the extent of skin fibrosis and internal organ involvement in dcSSc and may serve as an activity marker of disease [65]. These observations could also be supported by the strong polarization of Topo-I-specific T cells from SSc patients toward a pro-inflammatory Th17 phenotype, that is directly associated with the occurrence and progression of ILD [66]. Although anti-TopoI are markers of a severe and progressive disease, in daily clinical practice, not all patients with anti-Topo I antibodies show a rapidly evolving fibrosis and some patients experience only moderate skin and lung involvement [67]. This could be due to a different isotype expression of anti-TopoI: in fact, some authors reported that SSc patients with both IgG and IgM anti-Topo I more often experience disease progression compared to IgG positive but IgM negative anti-TopoI antibodies [68].

Various authors reported a relative higher risk for all sites malignancy in SSc [69]: although not confirmed by different cohorts, anti-Topo I have been reported as associated to higher risk of malignancy during SSc disease course, with a negative impact on survival [70].

### Anti-Th/To Antibodies

Anti-Th/To antibodies are directed towards several protein components of the RNase MRP complex [71], ubiquitously expressed endoribonuclease and often representing the target of autoantibodies in several autoimmune diseases. Almost all protein components have been reported as autoantibody targets in patients with rheumatic diseases, but Rpp25, Rpp38 and hPop1 are considered as the major autoantigens [20, 72]. Th/To antigens are located in the nucleolus of the cell and anti-Th/To antibodies recognize two endoribonucleases: the ribonucleoprotein 7–2/MRP-RNP (Th) and the ribonuclease 8–2/RNP (To) [73]. They produce a typical homogeneous nucleolar staining by indirect immunofluorescence (IIF) on HEp-2 cells, namely AC-8, according to ICAP nomenclature [51]. Despite being known for almost 40 years (first identified in 1982), these SSc autoantibodies have not been used much in clinical practice due to unavailability of antibody testing [14]. Anti-Th/To antibodies are fairly specific for SSc, because they are found in patients with SSc and primary Raynaud’s phenomenon, but not in those with systemic lupus erythematosus, polymyositis/dermatomyositis, or undifferentiated connective tissue disease. In addition to SSc, anti-Th/To autoantibodies have also been reported in rheumatoid arthritis and ILD [74, 75]. The specificity for SSc makes it an important serological tool in the diagnosis and stratification of SSc patients, especially given the fact that anti-Th/To are the most common antibodies found in patients who have tested anti-nuclear antibody (ANA) negative with widely available commercial assays (a false negative group of patients) [76].

The reported prevalence of anti-Th/To autoantibodies in SSc varies between 1 and 13% [19, 43, 77], with data suggesting a significant effect of ethnicity on autoantibody profile and clinical features (higher in Caucasian American SSc compared to African and Latin American SSc patients) [78].

In SSc, anti-Th/To antibodies have consistently been associated with lcSSc and ILD, defining a specific clinical pattern. In comparison to ACA, the main antibody associated with lcSSc, anti-Th/To positive SSc patients present shorter time between the onset of Raynaud’s phenomenon and the onset of SSc compared with ACA patients (< 2 years vs. > 2 years) [79]. Nevertheless, Th/To patients are not a risk group for developing diffuse cutaneous disease or renal impairment [17]. Moreover, they have less prominent skin changes, vascular disease and gastrointestinal involvement in comparison to ACA patients [17]. Similarly to other SSc-related autoantibodies, the association between Raynaud's phenomenon and anti-Th/To antibodies defines a pre-clinical SSc and presents an important risk factor for developing SSc [30]. In addition, Th/To patients present an earlier development of nailfold capillary microscopy abnormalities [30], are younger and more frequently male compared to ACA patients [19]. Anti-Th/To antibodies have also been associated with pericarditis in SSc patients [19].

Despite having more subtle initial presentation, anti-Th/To antibodies, in line with other nucleolar antibodies, present a high frequency of ILD, PAH, and myositis [17, 19]. Interestingly, anti-Th/To-positive patients usually develop both ILD and PAH. Pulmonary involvement is significantly more common in the anti-Th/To than in ACA+ patients (74% versus 51%, respectively) [17]. Anti-Th/To antibodies have been reported in almost 50% of ANA positive idiopathic pulmonary fibrosis patients [80]. Given the high incidence of lung involvement, survival of this group is decreased in comparison to other lcSSc patients [81]. Anti-Th/To has the lowest survival rate in limited SSc patients: cumulative 5- and 10-year survival rate (78 and 65%, respectively) [8].

Anti-Th/To autoantibodies are a relevant marker of SSc, most frequently associated with lcSSc and ILD. Their use in clinical practice is essential for better diagnostic distinction and stratification of SSc patients.

## A SSc-Specific Marker with New Perspectives: Anti-RNA Polymerase III Antibodies

Antibodies reactive with RNA polymerase III (anti-RNAP3) were first identified in 1993 [82, 83]. Even though they are a highly specific biomarker of SSc with relevant clinical associations [15, 83–85], their use in clinical practice was initially limited by the cumbersome procedures required by the immunoprecipitation (IP) assay used in the early investigations. The identification of the major antigenic site universally recognized by anti-RNAP3 positive SSc sera led to the development of sensitive and specific immunoassays, based on the ELISA or multiplex line immunoblot (LIA) methods [15, 16, 86–88]. This allowed the identification of previously unnoticed clinical features in anti-RNAP3-positive patients [85]. Although anti-RNAP3 seems to be very rarely associated with other SSc-specific antibodies (anti-TopoI, ACA), simultaneous positivity for other autoantibodies when analyzing SSc sera by LIA may represent a frequent issue [89]. In these cases, the clinicians should be guided by the presence of anti-RNAP3 in these patients, irrespectively from the presence of other autoantibodies. Although there are some indications that clinical correlations with anti-RNAP3 are stronger in patients with high antibody titer [89], there is currently no evidence for the clinical utility of monitoring anti-RNAP3 titer during follow-up.

Anti-RNAP3 are the most frequent antinuclear antibodies in SSc, after ACA and anti-TopoI: in a meta-analysis of 30 studies [90], overall pooled prevalence of anti-RNAP3 was 11% (95% CI 8–14). A high degree of heterogeneity between studies is at least partially explained by geographic factors: the prevalence of anti-RNAP3 among SSc patients is lower (3–10%) in Southern and Central Europe and Asia than in Northern Europe, North America, and Australia (15–22%) [90]. Few data about ethnicity are available: some American studies reported a lower frequency of anti-RNAP3 in African than in Caucasian patients [89], suggesting that ethnicity may contribute to explain the variability of anti-RNAP3 prevalence [90].

On the other hand, anti-RNAP3 are highly specific for SSc [15, 16, 88, 89]. Interestingly, during the development of the 2013 ACR/EULAR classification criteria for SSc, anti-RNAP3 were positive in 27/268 (10%) SSc patients in the criteria validation cohort, as compared with 0/137 in patients with a SSc-like disorder [13].

Consequently, anti-RNAP3 were included among SSc-related autoantibodies as a specific item in the 2013 SSc classification criteria. Therefore, there is a consensus that anti-RNAP3 can be helpful in the diagnosis and classification of SSc when a specific clinical suspect is present [13].

The utility of testing for anti-RNAP3 antibodies in patients with definite SSc derives from the peculiar clinical correlations of these autoantibodies. Testing for anti-RNAP3 therefore provide important information for patients’ management, indicating the need for a careful follow-up, particularly during the first phases of the disease, leading to early consideration of aggressive disease-modifying therapies in positive patients [85, 89].

### Association with Rapidly Progressive Skin Involvement and Joint Contractures

The association of anti-RNAP3 with the dcSSc subset is well known [15, 81–85]: in the EUSTAR registry, 58% of anti-RNAP3+ SSc patients had dcSSc compared with 28% anti-RNAP3- [13]. A peculiar aspect is the rapid progression of skin involvement among anti-RNAP3+ SSc patients: the time intervals between the onset of Raynaud's phenomenon and other symptoms, and between the first SSc-related symptom and the peak of (mRSS, are shorter in anti-RNAP3+ SSc patients than in patients with anti-TopoI or ACA [13, 59, 91–93]. Anti-RNAP3+ patients developing mRSS > 20 points did so within 3 years after onset of Raynaud’s phenomenon [59], and 90% of them reached their peak of mRSS within the first 2 years of the disease [13]. These data suggest that, after the first few years of the disease, skin involvement does not further progress in anti-RNAP3+ patients. Furthermore, in many patients, skin thickness may regress during the follow-up, even without treatment [59].

Despite possible skin involvement regression, anti-RNAP3+ patients suffer from a higher frequency of joint contractures [13] and may need early physical and occupational therapy to prevent or reduce this complication.

### Association with Scleroderma Renal Crisis

Anti-RNAP3+ SSc patients have the highest risk for developing SRC compared to other SSc groups [15, 82–85]: approximately 50% of patients with SRC have anti-RNP3 antibodies [13, 94–96]. However, case series from Japan and Italy show a lower incidence of SRC compared with the UK and North America, reflecting the lower prevalence of anti-RNAP3 antibodies [97–99]. Conversely, prevalence of SRC among anti-RNAP3+ SSc patients during the disease course ranges from 12 to 24% [13, 96]. The time interval from SSc onset to SRC is shorter in patients with anti-RNAP3 antibodies than in those with anti-Topo I [97, 99]. Close follow-up of renal function and blood pressure monitoring are mandatory in these patients.

### Association with GAVE (or “Watermelon Stomach”)

After the first small single-center report [100], different multicenter studies confirm the association between gastric antral vascular ectasia (GAVE) and anti-RNAP3+ , with an odds ratio of 4.6 [7, 101]. In a case–control study, clinically relevant GAVE was identified in 8% of anti-RNAP3+ vs. 1% of anti-RNAP3- patients [101]. Similarly to what has been reported for rapid progression of cutaneous involvement and SRC, GAVE was more frequently observed during the first phases of diseases [98, 99].

### Association with Cancer Synchronous to SSc Onset

The association of anti-RNAP3 antibodies with cancer diagnosis in close temporal relationship to SSc onset was first identified by a single-center American study [102] and confirmed by several single-center cohorts from different geographical areas [103–106] as well as by an international multicenter case–control study [92]. Anti-RNAP3+ SSc patients were reported to have an OR of 7.38 of having a cancer diagnosis within a short time frame around SSc onset, as compared with matched anti-RNAP3-negative patients [92]. The frequency of cancer diagnosis within 2 years before or after SSc onset in anti-RNAP3+ patients was 9% [92]. Breast cancers are the most prevalent type in females [92, 102], but other cancers may be found, particularly in males. Anti-RNAP3+ patients with older age or dcSSc are particularly at risk [92].

There might be a mechanistic link between cancer and the development of SSc [107]: this is due to the presence of genetic abnormalities in the RPC155/POLR3A gene in the cancer tissues from anti-RNAP3+ SSc patients (but not in cancers from other SSc patients), and of mutation-specific T-cell and B-cell immune responses cross-reacting with both mutated and wild-type RNAP3 protein [108].

Anti-RNAP3+ SSc patients may therefore benefit from cancer screening at the time of diagnosis [85]. The optimal approach for this screening may be guided by the recommendations proposed by EUSTAR experts [92].

Notably, women with breast cancer without rheumatic diseases do not carry anti-RNAP3, confirming the specificity of these autoantibody for SSc, and suggesting that it may be a cancer biomarker only in patients with this disease [109].

Finally, it should be remarked that no available data so far suggest that the risk of cancer in anti-RNAP3+ SSc is extended beyond a time interval close to disease onset [92, 103] and that the large majority of these patients do not have a detectable synchronous malignancy [92]. Anti-RNAP3+ SSc patients without cancer were shown to carry more frequently antibodies against a RNA-polymerase I large subunit (RPA194) [110].

### Possible Association with Silicone Breast Implants Rupture

Although previous epidemiological studies did not support a causal association link between silicone breast implants (SBI) and SSc, a recent analysis by the US Food and Drug Administration post approval studies, including nearly 100,000 individuals with SBI, described an increased rate of SSc as compared to normative data (standardized incidence ratio 7.00) [111]. In a single-center Japanese study evaluating 262 women with SSc, 4 out of 6 with SBI were anti-RNAP3+ . Moreover, in some cases, SBI had to be removed, suggesting a possible rupture of the implants as trigger of the autoimmune response [112]. In a multicenter Italian study, 11 SSc patients had a SBI implantation before SSc diagnosis. In the anti-RNAP3+ subset higher numbers of patients with SBI (*p* = 0.0002), SBI rupture (*p* < 0.0001), and SBI rupture in the absence of a history of breast cancer (*p* = 0.006) were recorded than in anti-RNAP3-negative patients, whereas no association with specific autoantibodies was found in patients with SBI without signs of rupture [113]. These observations, suggesting a link between SBI rupture and induction of anti-RNAP3+ SSc, indicate the need of further studies are to better define the characteristics of this syndrome and the possible effects of SBI removal and immunosuppressive treatment on its evolution [114].

### Other Clinical Associations (Interstitial Lung Disease and Pulmonary Hypertension)

Most cohort studies described no association between anti-RNAP3 and ILD. Indeed, rates of clinically significant ILD among anti-RNAP3+ are much lower (around 50%) than those seen in anti–TopoI+ patients [115]. However, in a prospective observational study, 18% of anti-RNAP3+ SSc patients developed extensive ILD [116], indicating that the issue cannot be neglected in all cases.

Anti-RNAP3 are generally not associated with PAH, but in the large single-centre cohort, these autoantibodies were shown to be an independent predictor of catheterization-proven PAH, either associated with ILD or not [62], but this observation should be verified in other, possibly multicenter, cohorts.

Finally, despite the high incidence of SRC and cancer synchronous to SSc onset, anti-RNAP3+ SSc patients do not show reduced survival as compared to the total cohorts of SSc patients [115].

## Markers of Overlap Syndromes

### Anti-Ku Antibodies

Anti-Ku antibodies were originally described in 1981, as serological markers of overlap Polymyositis/SSc (PM/SSc), but they later could be found in additional different connective tissue diseases [117, 118]. The Ku complex is a heterodimer made of p70 and p80 subunits that play several crucial biological functions as DNA repair, transcriptional regulation, and telomere activity [119]. The Ku protein is ubiquitously found both in the nucleus, in the cytoplasm or on cellular surfaces and in humans, the two subunits’ genes are localized on different chromosomes (22q13 and 2q33, respectively) [119]. In SSc, they are detected with a prevalence ranging from 2 to 6.8% and are significantly associated with PM/SSc overlap syndrome [118, 120]. In a European EUSTAR-initiated multicenter case-controlled study with 625 SSc patients, anti-Ku antibodies were found in 14 patients (2.2%), mainly associated with synovitis, joint contractures, and clinical features of myositis, and negatively associated with vascular manifestation of disease [120]. More recently, an international multicenter study included data of more than 2000 SSc patients, with 24 cases of anti-Ku [118]: Ku was present as a single specificity in 13 cases, whereas in the other cases it was associated with other specific or associated antibodies; ILD more frequently occurred in anti-Ku positive patients, and this association was also confirmed for the single specificity. Finally, in an Italian study, 13 out of the 46 Ku-positive patients presented a diagnosis of the SSc spectrum diseases (including PM/SSc, DM/SSc and SSc): all displayed a limited cutaneous involvement with a higher prevalence of myositis, arthritis, and ILD [121]. Anti-Ku is rarely found in SSc children or adolescents, and, in fact, only seven cases of overlap syndrome are described, all presenting with myositis and with only one case of ILD [122].

### Anti-Fibrillarin (U3-RNP) Antibodies

Anti-fibrillarin (U3-RNP) antibodies are directed against a highly conserved 34-kDa basic protein of the U3 small nucleolar ribonucleoprotein macromolecular complexes called fibrillarin [123–125], characterized by a clumpy nucleolar staining IIF pattern. Anti-U3RNP are detected in 5–14% SSc patients and in 95% of cases they are mutually exclusive from other SSc-specific autoantibodies [126–128]. Their presence is associated with a well-defined clinical phenotype: male patients, Afro-Caribbean descent, younger at diagnosis in comparison with other SSc patients and higher risk of developing PAH and gastrointestinal involvement, especially in African patients [126–128].

A recent French collaborative work [127] confirmed the association with overlap myositis: muscular involvement was more common in anti-U3RNP positive compared to negative patients, (31.4% versus 12.2%), with a prevalence of 50% among U3RNP positive patients with a diffuse cutaneous involvement.

### Anti-NOR90/hUBF Antibodies

Anti-nucleolar organizer regions (NOR) antibodies were initially described in patients with SSc in 1987 [129], targeting a novel 90 kDa nucleolar protein. In 1991, Chan et al. showed that the autoantigen recognized by human anti-NOR 90 autoantibodies is identical to human upstream-binding factor 4 (hUBF) [130]. These autoantibodies are a rare occurrence and are mostly associated not only with SSc but also with various autoimmune diseases [131]. In SSc, anti-NOR90 antibodies are usually reported in approximately 5% of patients, and they are mostly associated with lcSSc and have a favorable prognosis. In a Greek study, despite the low prevalence, they were strongly correlated with ILD [132].

### Anti-Ro52 Antibodies

Antibodies to Ro52 are the most common autoantibodies in different systemic autoimmune diseases: they are originally described as directed towards one protein of the cytoplasmic ribonucleoprotein complex including Ro60 and HYRNAs, but recently, Ro52 has been identified as the E3 ubiquity ligase, member of the tripartite motif (TRIM) family of proteins known as TRIM21 [133].

Anti-Ro52/TRIM21 antibodies were found in 20–27% of SSc in different multicenter cohorts [134, 135], representing the second most common autoantibody in SSc, overlapping with SSc-specific autoantibodies. Anti-Ro52 is known to be associated with anti-synthetase antibodies and frequently found in patients with IIM and ILD [136–138]. However, the clinical associations of anti-Ro52 in SSc have not yet been elucidated. Various authors report a significant association between anti-Ro52 and ILD in different SSc cohorts [134, 137, 138]. In an Australian study, anti-Ro52 antibodies are considered an independent prognostic factor for mortality in SSc (OR: 1.6), and a risk factor for PAH, independently from the occurrence of ILD [139].

### Anti-PM/Scl Antibodies

The PM/Scl autoantigen is a macromolecular complex, recognized as the human exosome, involved in RNA degradation and processing. The main autoantigenic proteins were named PM/Scl-75 and PM/Scl-100, based on their apparent molecular weights, but other exosome proteins were also proven to be target of autoantigens [140]. Antibodies against the PM/Scl complex are found in patients with SSc, PM, DM, and SSc/PM overlap syndrome [140].

Anti-PM/Scl antibodies show a nucleolar staining in IIF, but they can be identified by different immunoassays: the main clinical associations of anti-PM/Scl antibodies in SSc were observed irrespectively of the immunoassay used [27, 141–147].

In a recent analysis of more than 7000 SSc patients from the international EUSTAR dataset, confirming previous studies [7, 27, 146, 148], anti-PM/Scl were found in 4% of cases, but associated SSc-specific autoantibodies were also observed in half of them [149].

The clinical phenotype of anti-PM/Scl+ SSc patients is characterized by a higher frequency of myositis, ILD, calcinosis, and DM cutaneous signs, as compared with other SSc patients [141, 143, 145, 147, 149]. On the other hand, esophageal involvement and pulmonary hypertension (estimated by echocardiography) are less frequent [143, 149]. The higher frequency of calcinosis and telangiectasia was observed in patients positive for anti-PM/Scl-100 only as compared with those positive for anti/PM/Scl-75 [146, 149]. Notably, calcinosis is associated with anti-PM/Scl also in patients with PM and DM [150], suggesting that this feature could be related to the autoantibody regardless of the clinical setting.

The presence of muscle involvement among anti-PM/Scl+ SSc patients was found to be associated with increased frequency of heart, tendon and intestinal involvement, thus suggesting that it might represent the marker of a more severe disease phenotype [149].

Compared to other SSc subsets, anti-PM/Scl+ SSc patients have a lower risk of death in the first 10 years of the disease [115, 143], and a higher risk in the later phases [115]. The reduced risk of death can be related to the slow evolution of ILD in these patients [115]. Indeed, although frequent in anti-PM/Scl+ SSc patients, ILD in this subset has shown a good functional outcome at least when observed in the first 10 years after diagnosis [115, 145, 149].

Although some studies raised the hypothesis that anti-PM/Scl+ were associated with SRC or with cancer [151, 152]. The analysis of the large EUSTAR database and an ad hoc designed case–control study failed to confirm these possible associations [149]. Finally, anti-PM/Scl+ SSc patients demonstrated a low incidence of PAH and cardiac involvement [115, 149].

In summary, anti-PM/Scl+ SSc patients are characterized by a clinical phenotype including muscle involvement, cutaneous signs of DM, calcinosis, and ILD (with a favorable functional outcome in the first decade of the disease), which might be named “anti-PM/Scl syndrome.” This was proposed as a distinct subtype of myositis, particularly rich in extra-muscular features [147]. However, many patients with this phenotype also fulfil SSc criteria, thus suggesting that this syndrome represents an overlap between SSc and PM/DM, more or less polarized toward one end of the spectrum, according to the individual patient history.

## Novel Biomarkers

This section focuses on novel SSc autoantibody biomarkers and their use for diagnosis, establishing disease severity as well as potential target therapy, as summarized in Table 1.

**Table 1:** Overview of novel biomarkers in SSc and their characteristics

Autoantibody	Target	Function	Method	Positivity rate	Clinical associations (features and phenotype)
Anti-elF2B	elF2B	Initiation and regulation of protein synthesis	IP IP-WB (fine cytoplasmic speckled pattern on IIF)	1–2%	- dcSSc - ILD - Lung fibrosis - Overlap features with arthritis or myositis - Malignancy association?
Anti-RuvBL1/2	RuvBL1/2 double hexamer	DNA repair, chromatin remodelling, transcription, small nucleolar RNP assembly	IP (speckled staining on IIF)	1–2%	- Higher age at onset - Higher frequency in men - dcSSc - Myositis overlap - GI dysmotility - Severe myocardial complications
Anti-RNPC-3	Small nuclear ribonucleoproteins U11 and U12	Catalyze pre-mRNA splicing	IP (speckled nuclear staining on IIF)	3–5%	- dsSSc and lcSSc - Raynaud’s phenomenon - ILD - Severe pulmonary fibrosis - PAH - GI disease and esophageal dysmotility - Myopathy - Malignancy
Anti-BICD2	Bicaudal D protein (BICD2)	Transportation of various cargos to microtubules	IP	20–35%	- dcSSc - ILD - Inflammatory myopathy

*IP* immunoprecipitation, *IP-WB* immunoprecipitation and western blot, *dcSSc* diffuse cutaneous systemic sclerosis, *ILD* interstitial lung disease, *RA* rheumatoid arthritis, *GI* gastrointestinal, *lcSSc* limited cutaneous systemic sclerosis, *PAH* pulmonary arterial hypertension

### Anti-elF2B Antibodies

The eukaryotic translation initiation factor 2B (eIF2B) is a multi-subunit complex with an essential role in the regulation of protein synthesis in the cells. The complex is formed of five subunits (α-ϵ) assembled as a heterodecamer with two copies of each subunit [153]. It is one of the largest and more complex guanine nucleotide exchange factors (GEFs). Regulation of the co-stoichiometric expression of the eIF2B subunits serves as a vital control point within protein synthesis and regulates translation initiation in response to cellular stress. Dysregulation of eIF2B activity is associated with a number of pathologies, including neurodegenerative diseases, metabolic disorders, and cancer [154].

For the first time, in a study conducted in 2016, autoantibodies against elF2B were described in patients with SSc [155]. Anti-eIF2B antibodies were identified by using IP for confirming the presence of specific cytoplasmatic autoantibodies, in patients negative for known SSc-specific autoantibodies. The study reported the presence of this new autoantibody in 1–2% of SSc patients and none in control serum, highlighting the specificity of anti-elF2B for SSc. Furthermore, all positive patients were suffering from dcSSc and ILD, and some also presented overlap features of myositis or rheumatoid arthritis [155].

It is also interesting to underline that 58% of SSc patients have “abnormal” antibodies against Epstein-Barr virus (EBV) [156] which expresses proteins that target all of the elF2B subunits; thus, anti-eIF2B autoantibodies may be produced for EBV molecular mimicry [156].

Moreover, a British study conducted in 2018 investigated the prevalence and clinical associations of cytoplasmic autoantibodies in ANA-negative SSc patients, among which anti-elF2B. This study confirmed the association of these autoantibodies with SSc and lung fibrosis, data previously emerged from the study conducted in 2016, but in a larger and more representative SSc patient population [157].

The most recent data on prevalence and clinical significance of anti-elF2B in SSc derive from a 2020 study. This study confirmed data from previous studies about rarity of anti-elF2B (by identifying them in 2.5% of sera) and about the association with ILD and diffuse SSc. Moreover, it reported the presence of anti-elF2B in patients with lung cancer [158]. Further analyses should be conducted to assess a possible correlation between elF2B and the risk of developing neoplastic disease.

Another aspect that emerged is the crucial role of specific laboratory techniques, namely IP and IP-WB, to identify new and rare autoantibodies correlated to SSc, such as the anti-eIF2B [158].

In conclusion, anti-eIF2B positivity should be suspected when SSc patients are negative for ACA, anti-Topo I, and anti-RNAP3 antibodies but have positive cytoplasmic staining with negative ANA at IIF. The continuation of further studies will be essential to better delineate the cohort of patients with SSc in which anti-elF2B are present and to fully understand their clinical significance and possible therapeutic implications [158].

### Anti-RuvBL1/2 Antibodies

RuvBL1 and RuvBL2 are eukaryotic paralogous proteins from the AAA+ ATPase family. Forming ring-shaped heterohexamers for stability and having ATP-ase activity, they are essential in the formation of 2 complexes: the PAQosome and the INO80 family of chromatin remodelers [159].

This complex takes part in many cellular processes, such as transcription, DNA repair, chromatin remodeling, cell proliferation and small nucleolar RNP assembly [160]. RuvBL1/2 interacts with and stabilizes a diverse array of complexes, but the precise mechanisms underlying its essentiality is still a subject of research.

Autoantibodies directed against RuvBL1/2 complex represent a novel class of antibodies specific to SSc, identified for the first time in 2014 [161].

Anti-RuvBL1/2 antibodies are detected in a small number of patients with various connective tissue diseases (or autoimmune hepatitis (AIH) by ELISA using a recombinant RuvBL fragment expressed in *E. coli* [162]. However, these antibodies were distinct from the anti-RuvBL1/2 detected in SSc patients by IP assay. Indeed, SSc-specific autoantibodies recognize the epitopes present on the native RuvBL1/2 complex, while the autoantibodies detected in patients with other connective tissue diseases react with epitopes present on recombinant rRuvBL1 and/or rRuvBL2 fragments, but not on the native complex. It has been shown that the immunoprecipitated RuvBL1/2 complex maintains its reactivity even after adsorption with antibodies against rRuvBL1 or rRuvBL2 [162].

Recent Japanese and North American studies with large cohorts revealed a very low prevalence, approximately 1–2% of these autoantibodies, and their association with a specific clinical phenotype of SSc. Similarly to autoantibodies against PM/Scl and Ku, anti-RuvBL1/2 are associated with SSc/myositis overlap, but anti-RuvBL1/2 positive overlap patients were found to have more frequently gastrointestinal and heart involvement than those with anti PM/Scl or anti Ku. Beyond that, anti-RuvBL1/2 antibodies also correlate with a unique SSc phenotype, associated with older age at SSc onset, male gender and diffuse skin thickening [157, 161].

### Anti-U11/12 RNP (RNPC-3) Antibodies

Small nuclear ribonucleoproteins (RNP) are well-known targets of the autoimmune response in a number of connective tissue diseases, including SSc. The primary target of these autoantibodies is the protein component of the complex. They are directed towards both discontinuous and linear epitopes which are either contained in the protein sequence or are post-translationally modified. Some recognize individual RNPs (i.e. U1, U3) while others act directly against a complex of RNPs [163].

The minor spliceosome complex consists of several small nuclear RNAs and multiple protein structures. U11/U12 RNPs, a component of spliceosome found in low abundance in eukaryotic cells, catalyze pre-messenger RNA (mRNA) splicing of nuclear pre-mRNA introns [164]. Formation of the U12-type pre-spliceosome is initiated by association of the U11 and U12 snRNPs, which, in contrast to U1 and U2, bind as a stable preformed di-snRNP. U11 and U12 recognize the splice site and the branch site, respectively, in a cooperative manner [164].

Anti-U11/U12 RNP (namely anti-RNPC3) antibodies were first described in 1993 by Gilliam and Steitz [165]. A study conducted at the University of Pittsburg in 2009 was the first to investigate the implication of these autoantibodies in clinical presentation of SSc. They reported the overall prevalence of anti-U11/U12 RNP autoantibodies at 3.2% and no association with other SSc-associated antibodies [163]. The most significant difference encountered between U11/U12 RNP positive and negative patients was the prevalence of pulmonary fibrosis, Raynaud’s phenomenon, and gastrointestinal (GI) involvement [136].

Furthermore, a recent North American study evaluated the association between anti-U11/U12 RNP antibodies and GI dysmotility and its severity. The data show a clear association between these autoantibodies and the presence of moderate to severe GI disease as well as esophageal dysmotility, alongside a high rate of ILD [166, 167]. This discovery indicates that anti-U11/U12 RNP antibody is a marker of great clinical importance in SSc.

Given that the patients with SSc present an elevated risk of cancer when compared to the general population [168], Shah et al. recently sought to verify the existence of any correlation between anti-RNPC3 and cancer [169]. The study determined that the presence of anti-U11/U12 RNP autoantibodies in a subset of patients with an increased risk of cancer at the time of the first clinical manifestations of SSc [169]. These antibodies are specific to SSc and are not found in cancer patients without SSc. Consequently, a hypothesis of cancer-induced autoimmunity can be made as well as suggest a targeted malignancy screening at SSc onset.

Compared with other known autoantibodies such as anti-RNAP3 and ACA, it emerged that both patients with anti-RNAP3 and anti-U11/U12 RNP present a mean age of SSc onset above 50 years and a shorter time to presentation for clinical evaluation than other antibody subgroups [169]. It is likely due to the aggressive phenotype associated with these two autoantibodies. Anti-U11/U12 RNP positive patients typically present with moderate cutaneous disease, are prone to severe restrictive lung disease at baseline (lower FVC and DLCO) and PAH, important GI involvement as well as severe Raynaud’s phenomenon and history of myopathy.

In conclusion, search for anti-RNPC3 antibodies is important to consider, especially in ANA-positive patients with speckled pattern and negative for other SSc-specific antibodies, in order to improve the serological diagnosis and be proactive in regards of treatment.

### Anti-BICD2 Antibodies

Cytoplasmic dynein is a large multiprotein complex, which is responsible for transporting various cargos to microtubules. Dynactin, another multiprotein complex, was found to be necessary for a large number of different types of dynein transport. It has been shown to improve dynein processing and to mediate its interaction with different cellular structures [170].

Bicaudal D protein (BICD) was initially identified as a component of dynein pathway in Drosophila, where it encodes for a protein involved in the correct localization of mRNA in Drosophila embryos. In humans, there are two homologs of BICD, named BICD1 and BICD2, that are highly conserved dynein motor adaptors. In particular, BICD1 and BICD2 consist of three coiled-coil segments separated by highly flexible regions, which are involved in microtubule-based transport [171].

Recent studies identified antibodies against human protein bicaudal D homolog 2 (BICD2) as a novel biomarker of SSc, as it was detected in patients negative for known SSc-specific autoantibodies.

The prevalence of anti-BICD2 antibodies ranges from 20 to 35% in SSc and from about 5–10% in controls [172, 173].

BICD2 autoantibodies target a unique linear epitope that is shared with CENP-A [174], but in addition to the BICD2 epitope targeted by BICD2/CENP-A cross-reactive antibodies, anti-BICD2 antibodies also recognize an epitope specific for the anti-BICD2 response [175].

Considering the correlation between the sequences recognized by anti-BICD2 and anti-CENP-A, it is not surprising to find SSc patients positive for both anti-BICD2 and ACA with the disease phenotype, similar to that of patients with ACA alone, characterized by lcSSc and PAH. However, the single specificity for anti-BICD2 antibodies is associated with a unique phenotype of SSc, related to the development of ILD, inflammatory myopathy, and dcSSc [172].

In conclusion, anti-BICD2 could be added to the list of SSc-associated antibodies capable of identifying a clinical profile characterized by ILD and inflammatory myopathy in SSc [172].

Further studies are needed to better delineate the prevalence and clinical significance of BICD2 antibodies in patients with SSc. Moreover, subjects with single specificity for BICD2 should be examined in depth in order to minimize the bias introduced by the presence of other SSc-specific antibodies.

## SSc Patients Negative for Autoantibodies

ANA are detected in more than 90% of SSc patients [1]. The exact mechanisms of ANA production and their role in the pathogenesis of SSc is still unclear. Nevertheless, identification of SSc-associated autoantibodies is essential as they are shown to have a considerable value in diagnosis and in predicting clinical outcomes.

A North American study published by Salazar et al. was the first to analyze in detail the demographic and clinical features of ANA-negative SSc patients in a large cohort [31]. The only demographic difference found between ANA-positive and ANA-negative group was the prevalence of male gender in ANA-negative patients. This finding underlines the importance of exploring more in detail the link between autoantibody production and genetics in SSc [6].

Only a few other reports have previously studied some of the clinical features of ANA-negative patients. In accordance with the results from a study conducted by Hamaguchi et al. [55] as well as the German network for SSc [43], Salazar et al. found that ANA-negative patients less likely present vasculopathic features of the disease such as digital ulcerations, telangectasia as well as PH [31]. Moreover, it is important to note the higher frequency of malabsorption as well as higher likeness to develop SRC encountered in ANA-negative population then in ACA or anti-TopoI positive patients [31]. Only RNAP3, a well-known risk factor for SRC, is associated with higher risk of developing SRC [176]. Furthermore, Salazar et al. observed more diffuse skin involvement in the ANA-negative group but interestingly found a lower fibrosis severity [31].

In conclusion, ANA-negative SSc patients comprise a subset of SSc patients requiring further understanding and evaluation. To date, they identify a subset of disease with specific characteristics, but it is likely that this negativity is only apparent and that new antibodies, including those discussed here, may actually be the biomarkers of this subset of SSc.

## Significance of SSc-Associated Autoantibodies in Localized Scleroderma

Autoantibodies have been described also in different types of localized scleroderma diseases, in particular in morphea. Morphea in a complex disease, occurring in both adults and children, traditionally been subdivided into different clinical subtypes: plaque, limited, generalized, bullous, linear, and deep morphea [177, 178]. Recently, morphea is not considered as “just a skin disease,” but it is widely accepted as a true autoimmune disease with circulating autoantibodies. A recent meta-analysis confirms the occurrence of ANA in about 6–68% of pediatric onset morphea patients, in particular when affected by linear morphea type [179]. ANA were reported also in adult patients, with different IFI pattern, namely speckled [180] or homogeneous [181], probably due to a reactivity with nucleosome and histones [182, 183].

ANA-positivity seems to associate with disease severity, represented by depth of skin involvement, extra-cutaneous manifestations, and probability of the disease flare after remission [184, 185].

Different authors, including wide morphea cohorts, reported SSc-associated autoantibodies positivity in a significant rate of patients: these autoantibodies did not predict the onset of SSc, but correlated with morphea disease severity, such as joint contractures, musculoskeletal involvement, higher skin thickness [179], or disease relapse [185]. Other non-SSc autoantibodies have been described, as rheumatoid factor, anti-ssDNA or anti-histone, and are considered markers of severe cutaneous disease [179]. Although the high rate of occurrence, ANA or SSc-associated autoantibodies are not considered a biomarker of localized scleroderma to date. Nevertheless, a dysregulation of immune system and fibrotic pathways seem to play a significant role in the pathogenesis of this disease [186].

## Conclusions

Even if the availability of validated and standardized immunoassays for the detection of classical autoantibody markers of SSc, the search and validation of novel autoantibodies is important for different reasons: we still have several diagnostic gaps in the diagnosis of SSc, in particular in seronegative SSc patients. Autoantibodies with high diagnostic specificity and high predictive values are required for early SSc diagnosis, for a specific follow-up and to define the best therapy for specific SSc subsets. In addition, novel serological biomarkers of SSc need to be validated on bigger cohorts in different ethnic groups. Furthermore, the use of different technology platforms for autoAb analysis, including the new and promising ones, requires the harmonization of results towards the development of standards and the characterization of reference materials that should ideally be homogeneous, stable, traceable, safe, ethical, available, and certified.

## Data Availability

The datasets used and/or analyzed during the current study are available from the corresponding author on reasonable request.
